# Periorbital Richter Syndrome

**Published:** 2015-06-01

**Authors:** Mark Gorman, Julia Ruston, Sarath Vennam

**Affiliations:** ^a^Division of Plastic Surgery, Royal Devon and Exeter Hospital, Exeter, Devon, United Kingdom; ^b^Division of Plastic Surgery, Royal Free Hospital, London, United Kingdom

**Keywords:** Richter syndrome, CLL, eyelid, necrotizing fasciitis, diagnosis

## DESCRIPTION

A 70-year-old with a background of chronic lymphocytic leukemia (CLL) and progressive necrotic cutaneous periorbital lesions was incorrectly managed as having necrotizing fasciitis (NF) when referred to plastic surgery. The underlying pathological process of Richter syndrome was missed until it was too late for this patient. Richter syndrome is a transformation of CLL into higher-grade lymphomas, driven by viral infections, whose successful treatment is dependent on rapid diagnosis and aggressive targeted chemotherapy/immunomodulation. It more commonly presents with lymphadenopathy but may, as in this case, present as a necrotic cutaneous basin, being easily mistaken for other differential diagnosis.

## QUESTIONS

1. **What is Richter syndrome?**
2. **How is Richter syndrome diagnosed?**
3. **How do you distinguishing between necrotic cutaneous differential diagnoses in patients with CLL?**
4. **How should Richter syndrome be managed with cutaneous presentations?**

## DISCUSSION

Richter syndrome is the transformation of CLL into diffuse, large B-cell lymphoma.^1^ Transformation into a more malignant phenotype is thought to be driven by viral infection, such as with Epstein-Barr virus or herpes simplex virus, as was cultured from wound swabs in this case.^2^ Richter syndrome affects 1 in 10 patients with CLL and is treatable, but it has a poor prognosis, median survival being 10 months from diagnosis.^3^ It presents with lymphadenopathy, hepatosplenomegaly, and raised serum lactate dehydrogenase levels.^3^ Extranodal manifestations can occur, with ocular, renal, and neurological systems affected.^4^

**Figure 1:**
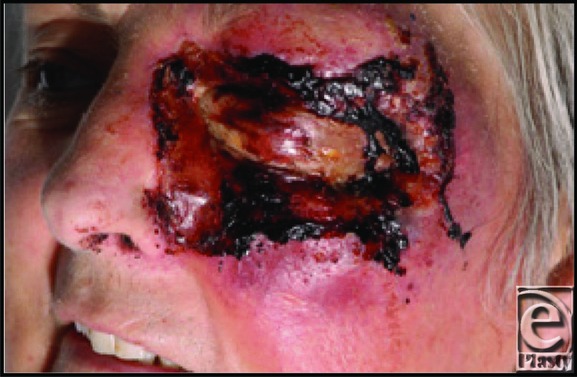
Richter syndrome affecting eyelids on the left side in our patient.

Once suspected, Richter syndrome (ie, background of CLL, worsening symptoms, and evidence of viral infection) is diagnosed by demonstrating CLL's histopathological phenotypic change through biopsy of the affected lymph, bone marrow, or as in this case, cutaneous involvement.^5^ Managed first by hematology, our patient presented with a small pedunculated pink lesion on the lower left eyelid with spreading erythema. Over a 3-month period, small areas of developing eyelid necrosis (Fig 1) were treated for suspected fungal, viral, and bacterial infections. Despite the background of CLL and being positive for herpes simplex virus, without an early biopsy, the basin of eyelid CLL and its transformation to a higher grade of lymphoma were missed. The patient was referred to plastic surgery for assessment of potential NF.

**Figure 2:**
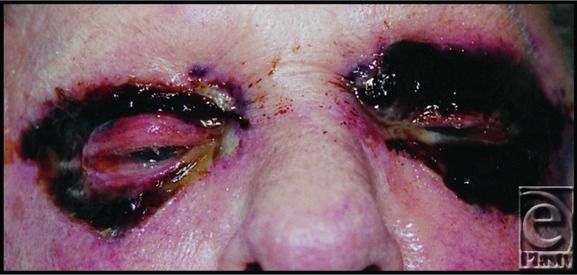
Necrotizing fasciitis affecting both eyes.

In a patient with CLL and cutaneous necrosis of the orbit, additional to the more common fungal and infective causes, although NF is less common, as an aggressive potentially deadly soft-tissue/fascial infection, it should be considered and either ruled out or treated.^6^ The necrosis associated with NF (Fig 2) is, on examination, difficult to distinguish from that of Richter syndrome (Fig 1). Clinically, suspicion of both NF and Richter syndrome would be raised by clinical deterioration, but NF is expected to have a more acute (hours as opposed to days) time frame. The blistering, necrosis, and potential cutaneous thrombosis observed would not tend to demarcate into the more stable pattern, as seen with Richter syndrome, where the area affected corresponds to the basin of cutaneous CLL as opposed to progressive soft-tissue/fascial infection. The distinguishing factors included the more indolent course of Richter syndrome, as compared with NF, a failure to respond to antifungal/viral/bacterial therapies, and the need for an early biopsy. Conversely, on the basis of reviews of periorbital NF, using the rate of necrotic progression, or whether lesions demarcate, may not be a wholly reliable way to distinguish it from Richter syndrome. Luksich et al^7^ somewhat controversially report that ocular NF presents less aggressively than in the rest of the body and may be treated conservatively. Given such presentations, the diagnostic suspicion of NF in this case is more understandable, but actually confounds the choice not to biopsy, prior to debridement. This error was made because of a lack of awareness of Richter syndrome and communication between the hematology and surgical specialties.

Not only is Richter syndrome frequently a difficult diagnosis to establish but it also presents a therapeutic challenge. Treatment of Richter syndrome is primarily through the administration of monoclonal antibodies, chemotherapy, and steroids in the acute care setting.^8^ Radiotherapy may be also be used in combination to control pain, lymphadenopathy, and neurological/spinal cord manifestations. Standard chemotherapies used in CLL are not effective, and most clinicians rely on combined CHOP-R therapy for diffuse large B-cell lymphoma: cyclophosphamide, doxorubicin, vincristine, prednisone, and rituximab.^8^ With complete remission difficult to achieve, the use of allogeneic stem-cell transplantation has shown early promise but is still in its infancy. The choice of the plastic surgery team to debride down to orbicularis was likely to have only added insult to an already immunocompromised patient, where prompt institution of steroids, targeted monoclonal antibodies, and chemotherapy would have given the patient the best outcome.
